# A 1D [Ni(L)(H_2_O)_3_]_n_·nH_2_O Coordination Polymer as a Dual Function Material for Antibiotic Detection and Dye Photo-Degradation

**DOI:** 10.3390/molecules30224366

**Published:** 2025-11-12

**Authors:** Fengli Yu, Mingxuan Zhu, Xiaoyu Weng, Dazhi Sun, Xingyuan Yu, Jiazhen Shi, Zhifang Liu, Xiaoyang Yu

**Affiliations:** 1 Jilin University of Chemical Technology, Jilin 132022, China; fengliyu@jlict.edu.cn (F.Y.);; 2 Jilin City Talent Service Center, Jilin 132022, China

**Keywords:** nickel-based coordination polymer, photocatalytic property, methylene blue, electrochemical detection, differential pulse voltammetry, norfloxacin

## Abstract

The development of materials for the remediation and monitoring of water environments remains a significant challenge in the field of environment and materials science. In this study, a nickel-based coordination polymer, [Ni(L)(H_2_O)_3_]_n_·nH_2_O (**1**), was synthesized employing 4,4′-(1H,1′H-[2,2′-biimidazole]-1,1′-diyl)dibenzoic acid (H_2_L). Single-crystal X-ray diffraction analysis showed that L^2−^ ligands connect Ni^2+^ ions into 1D Z-shaped chains via two coordination modes. The chains are further assembled into a 3D supramolecular structure through hydrogen bonding interactions. The photocatalytic test showed that complex **1** could effectively degrade the organic dye methylene blue (MB). Under the conditions of catalyst dosage 5 mg, MB initial concentration 20 ppm and pH 7, the degradation efficiency reached 87.7% within 180 min. In addition, complex **1** can be used for the electrochemical detection of norfloxacin (NOR) by differential pulse voltammetry (DPV), exhibiting a linear response in the concentration range of 2–197 μM and the detection limit (LOD) of 1.74 μM. These results demonstrate that complex **1** has bifunctional properties of photocatalytic degradation of organic dyes and electrochemical sensing of antibiotic NOR, making it a promising candidate material for the synergistic treatment of complex pollutants.

## 1. Introduction

With the rapid development of industrialization, water pollutants, such as organic dyes and antibiotics, have become major global environmental problems, causing harm to all living organisms, including humans, by affecting health and living conditions [1,2]. Owing to the stable chemical properties and poor biodegradability, these pollutants exhibit significant resistance to removal and present considerable challenges for accurate trace-level detection [3,4]. The development of efficient technologies for treating refractory organic dyes and the design of high-performance sensing materials for detecting trace antibiotics in aqueous environments represent two current research hotspots in environmental and materials sciences [5,6]. Among these, solar-driven photocatalytic degradation technology and novel electrochemical sensors represent two efficient, environmentally friendly, and economical technical approaches for water pollution control [7,8].

Coordination polymers (CPs) exhibit excellent performance in photocatalytic degradation and electrochemical treatment of pollutants in aqueous environments [9]. The variable-valence metal centers in CPs act as electron transport nodes, facilitating efficient electron transfer through reversible redox cycles. Under visible-light irradiation, these centers not only promote interfacial charge separation but also suppress the recombination of photogenerated electron–hole pairs, thereby enhancing the overall efficiency of the photocatalytic system [10]. For instance, the team of Krushika Mhalshekar synthesized a BC@Co-MOF via in situ growth of Co-MOF on bacterial cellulose (BC). Moreover, it exhibited the highest photocatalytic degradation rates of 92.52% for Malachite Green (MG) and 82.06% for Cr(VI) [10]. The electron transfer capability of the metal centers endows the CPs with excellent electrochemical performance for detecting trace pollutants in aqueous environments [11]. For example, Ruifang Xiang’s group successfully synthesized two new cobalt(II)-based CPs, namely ([Co(HL)(bix)]·H_2_O)_n_ (1) and ([Co(HL)(bimb)]·H_2_O)_n_ (2), (H_3_L = 2,4,6-tris(4-carboxyphenyl)-1,3,5-triazine, bix = 1,4-bis(imidazol-1-ylmethyl)benzene, and bimb = 1,4-bis((1H-1,2,4-triazol-1-yl)methyl)benzene). When employed as electrochemical sensors for ciprofloxacin detection using differential pulse voltammetry (DPV), the two CPs exhibited linear detection ranges of 2–20 μM and 1–14 μM, with LODs of 0.135 μM and 0.082 μM, respectively [12].

However, the development of CPs that can simultaneously address these two types of technical issues through a single material system remains a significant challenge in the field of environmental science, with relevant research remaining relatively scarce.

The synergistic enhancement of both photocatalytic and electrochemical properties in CPs can be achieved through the rational selection of metal centers and organic ligands, coupled with control over their crystal structures. Research has demonstrated that transition metals can significantly regulate essential properties of coordination polymers, such as light absorption, charge separation efficiency, and electrical conductivity [13]. Nickel-based CPs have become ideal multifunctional materials, owing to excellent structural stability, efficient electron transfer regulation, and outstanding catalytic activity [13]. Furthermore, the rational design of organic ligands is also the key to regulate and optimize these multifunctional properties. Aromatic compounds are often selected as ligands not only due to their rigid molecular skeletons that confer structural stability, but also because of their rich π-electron systems [14]. The delocalized π-electrons in these aromatic rings facilitate electron donation and redistribution, which significantly enhances the optical properties of the coordination polymers [14,15]. For instance, the team of Tianrui Qin selected 2,4,6-tris(4-carboxyphenyl)-1,3,5-triazine (H_3_L) and 1,4-bis(imidazol-1-yl)benzene (bib) to synthesize [Ni(HL)(bib)_0.5_·2H_2_O]ₙ (1) and [Ni(HL)(bib)_1.5_]ₙ (2). The chemical sensors fabricated from CPs 1 and 2 exhibited detection limits for ciprofloxacin (CIP) of 0.057 µM and 0.014 µM, respectively, as determined by DPV [16]. Jing-Wang Cui’s team has prepared a nickel-based CP, [Ni(1,4-bib)_1.5_(TPA-Cl_2_)·H_2_O]_n_, (1,4-bib = 1,4-bis(1H-imidazol-1-yl)benzene, H_2_TPA-Cl_2_ = 2,5-dichloro-terephthalic acid), and this CP exhibited photocatalytic activity for the degradation of organic dyes, achieving a degradation rate of 85.7% for Rhodamine B (RhB) in an aqueous system [6].

Based on the above considerations, in this work, a new 1D nickel-based coordination polymer, [Ni(L)(H_2_O)_3_]_n_·nH_2_O (**1**), was synthesized employing H_2_L (H_2_L = 4,4′-(1H,1′H-[2,2′-biimidazole]-1,1′-diyl)dibenzoic acid). Photocatalytic experiments demonstrated the efficacy of complex **1** in degrading methylene blue (MB). In addition, a sensor fabricated from **1** exhibits a low detection limit toward trace norfloxacin (NOR) by DPV.

## 2. Results and Discussion

### 2.1. Syntheses Discussion

In hydrothermal synthesis, the nucleation and crystal growth of the target product are influenced by multiple factors, including reactant concentration, pH of the system, and reaction time, etc. [17]. For the formation of complex **1**, the pH value was found to be particularly critical. When the pH value was below 5.5 or above 7.0, no defined crystals were obtained, resulting instead in light green powder. Furthermore, when the reaction proceeded for less than 3 days, only small and irregular crystals were produced.

### 2.2. Description of Crystal Structure

**Figure 1:**
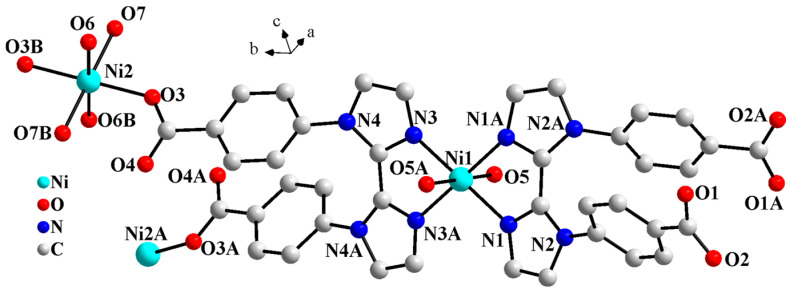
Molecular structure diagram of **1** (symmetry codes: A, 1 − x, y, 0.5 − z; B, 1 − x, 2 − y, 1 − z).

Crystallographic analysis indicates that complex **1** features 1D Z-shaped chain, crystallizing in monoclinic space group *C*2*/c*. The asymmetric unit of complex **1** consists of two Ni^2+^, two halves of L^2−^, three coordinated and one lattice water molecules. As shown in Figure 1, Ni ions are six-coordinated octahedral geometries in two different coordination environments. Ni1 is coordinated by four nitrogen atoms from two L^2−^ (N1, N1A, N3, and N3A, symmetry code: A, 1 − x, y, 0.5 − z) and two coordinated water molecules (O5 and O5A). Ni2 is coordinated by two carboxylate oxygen atoms from two L^2−^ (O3 and O3B, symmetry code: B, 1 − x, 2 − y, 1 − z) and four coordinated water molecules (O6, O6B, O7 and O7B).

**Figure 2:**
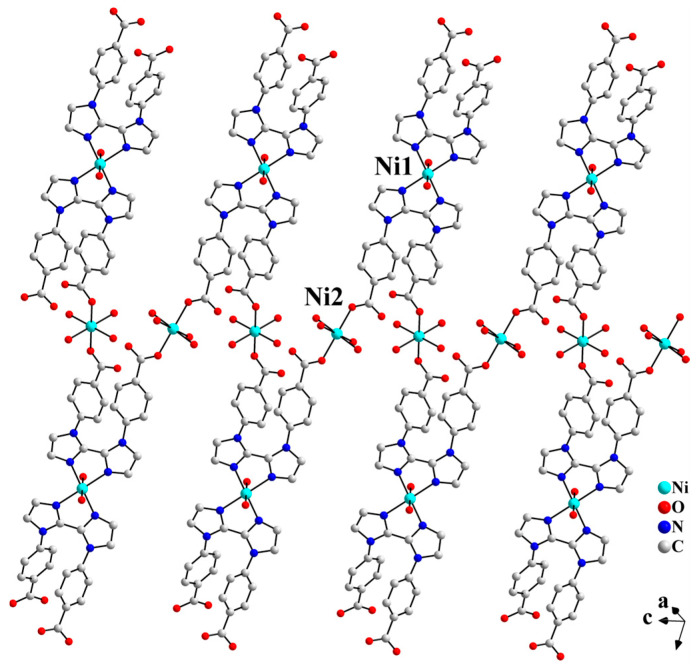
The 1D Z-shaped chain structure of **1**.

As shown in Figure S1, in complex **1**, the L^2−^ ligand exhibits two coordination modes: *μ*_3_-*k*N,N′;*k*O;*k*O′ and *μ*-*k*N,N′. The *µ*_3_-L^2−^ ligand chelates Ni1 through two biimidazole nitrogen atoms (N3 and N3A) and simultaneously bridges Ni2 and Ni2A via two carboxylate oxygen atoms (O3 and O3A), resulting in a 1D Z-shaped chain (Figure 2). Meanwhile, the *μ*-L^2−^ ligand further chelates Ni1 on both sides of the Z-shaped chain through the diimidazole nitrogen atoms (N1 and N1A), thereby stabilizing the chain. The two carboxylic groups of the *μ*-L^2−^ ligand are both deprotonated. However, none of the four carboxylate oxygen atoms (O1, O1A, O2, and O2A) coordinate to the Ni centers. Liu et al. reported a Pb-based CP constructed from the H_2_L ligand, where the organic ligand displays a *μ*_3_-*k*N;*k*O,O′;*k*O″,O‴ coordination mode. As shown in Figure S1c, the carboxylic groups of the two benzoate moieties chelate the Pb(II) ions. However, due to steric hindrance, the two imidazole rings of the biimidazole unit are twisted at a certain angle. As a result, only one imidazole coordinates to the Pb(II) ion via its nitrogen atom [18].

**Figure 3:**
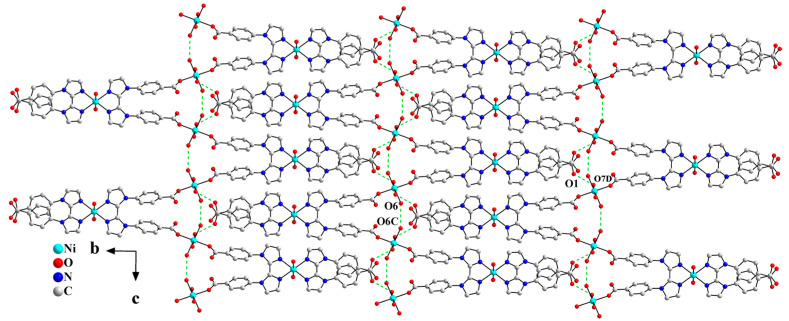
One-dimensional Z-shaped chains are extended into a 2D layered structure in the *bc* plane via hydrogen bonding interactions (symmetry codes: C, 1 − x, y, 1.5 − z; D, 1 − x, 1 − y, 1 − z).

There exist intramolecular hydrogen bonding interactions between coordinated water molecules O6 and O6C (symmetry code: C, 1 − x, y, 1.5 − z) within the 1D Z-shaped chain. Adjacent Z-shaped chains are further extended into a 2D supramolecular layered structure through the intermolecular hydrogen bonding interactions between the carboxylate oxygen atom O1 and the coordinated water molecule O7D (symmetry code: D, 1 − x, 1 − y, 1 − z), as shown in Figure 3.

**Figure 4:**
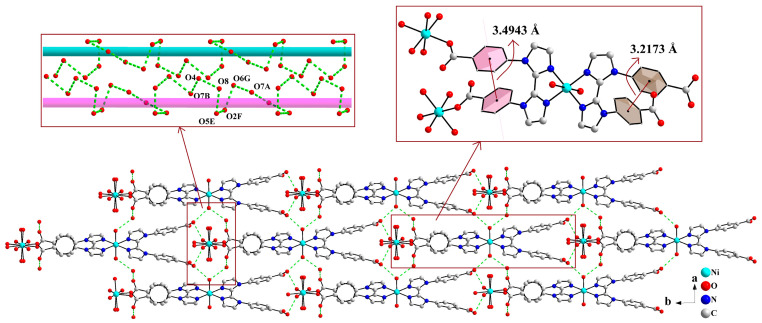
Two-dimensional supramolecular layers are linked into a 3D supramolecular network structure through weak interactions (symmetry codes: E, −0.5 + x, 0.5 + y, z; F, x, 1 + y, z; G, x, 2 − y, −0.5 + z).

As shown in Figure 4, the supramolecular layers are linked into a 3D supramolecular structure by chains of hydrogen bonding interactions among carboxylate oxygen atom, coordinated water molecules, and lattice water molecules (O7A···O6G···O2F···O5E···O8···O4···O7B, symmetry codes: E, −0.5 + x, 0.5 + y, z; F, x, 1 + y, z; G, x, 2 − y, −0.5 + z). In addition, π···π stacking interactions occur between adjacent benzene rings of L^2−^ ligand, with centroid-to-centroid distances of 3.4943 Å and 3.2173 Å, respectively. These hydrogen bonding and π···π interactions collectively stabilize the 3D supramolecular structure.

### 2.3. Powder X-Ray Diffraction, FT-IR Spectra and TG Analyses

As shown in Figure S2, the experimental powder X-ray diffraction (PXRD) spectra of complex **1** are in close agreement with the simulated spectra derived from the single crystal data, confirming the phase purity of the sample [19]. The difference in the preferred orientation of the powder sample may be the cause of the intensity discrepancy between the experimental and simulated PXRD patterns [20].

Figure S3 shows the infrared spectra of free H_2_L ligand and complex **1**. As shown in Figure S3b, complex **1** exhibits a broad band peak at around 3230 cm^−1^, which can be attributed to the O-H stretching vibrations of the coordinated and lattice water molecules [21]. Compared with the free H_2_L ligand, the peaks at 1606–1394 cm^−1^ should be attributed to the completely deprotonated carboxylate groups and the C=N stretching vibration of the imidazole groups of the organic ligand [22,23]. The coordination mode of the carboxylate group can be determined by the value of Δν between the asymmetric and symmetric stretching vibrations (Δν = ν_as_(COO^−^) − ν_s_(COO^−^)). When Δν > 200 cm^−1^, the carboxylate group is either free or coordinates to the metal in a monodentate fashion; while Δν < 200 cm^−1^, the carboxylate group adopts chelating coordination mode to the metal [24,25]. In complex **1**, the Δν value of 212 cm^−1^ is consistent with the single-crystal X-ray diffraction analysis, which reveals that the carboxylate groups of the *μ*_3_-L^2−^ ligands adopt a monodentate coordination mode, whereas those of the *μ*-L^2−^ ligands remain fully uncoordinated. Additionally, in the spectrum of complex **1**, the characteristic bands observed around 1100 cm^−1^ are attributed to the in-plane bending vibrations of the benzene ring [26], and those in the 510–420 cm^−1^ range are assigned to Ni–O and Ni–N vibrations [3].

In an air atmosphere, the thermal decomposition process of complex **1** proceeds two-step weight loss (Figure S4). The first weight loss stage (50–209 °C) corresponds to the removal of one lattice water molecule and three coordinated water molecules (found, 13.94%; calcd., 14.31%). The second weight loss stage (359–454 °C) is attributed to the decomposition of the organic ligand (found, 71.99%; calcd., 73.94%). The final residue belongs to NiO, with a residual mass of 14.07%, in agreement with the theoretical value (14.85%). Differential scanning calorimetry (DSC) of complex **1** reveals an endothermic event with an estimated enthalpy change (ΔH = 543.3 kJ mol^−1^) in the temperature range of 50–209 °C, corresponding to dehydration. In contrast, a strong exothermic process is observed in the range of 359–454 °C with an estimated ΔH of –5634.7 kJ mol^−1^, which is attributed to oxidative decomposition of the organic ligand in an air atmosphere [27,28].

### 2.4. Photophysical Properties of Complex ***1***

The band gap energy (Eg) of complex **1** was determined from the solid diffuse reflectance spectroscopy. As shown in Figure S5a, complex **1** exhibits strong absorption in the ultraviolet region (200–400 nm). The strong absorption in this region usually corresponds to ***π*** → π* electronic transitions in the material, which may be attributed to the conjugated structure of the aromatic rings of the organic ligand [29,30]. The reflectance data were converted to absorption data using the Kubelka-Munk function, and the Eg value of 3.3362 eV was obtained by extrapolating the linear region of the absorption edge [21]. This indicates that **1** exhibits semiconductor behavior, suggesting its promise as a photocatalytic material [31].

As shown in Figure S5b, the Mott-Schottky (M-S) plot of **1**-GCE exhibits a negative slope, indicating p-type semiconductor behavior [32,33,34,35]. The flat band potential (E_FB_) of **1** was measured to be 0.2674 V. This value was converted to the standard hydrogen electrode (NHE) scale using the equation E_NHE_ = E_Hg/Hg2Cl2_ + 0.242 V, yielding a final E_FB_ of 0.5094 V (V s. NHE) [36,37].

As shown in Figure S6, the semiconductor photoelectrode of complex **1** (**1**-SP) exhibits a photocurrent response, evidencing efficient photogeneration, migration, and separation of charge carriers [38,39]. Electrochemical impedance spectroscopy (EIS) was employed to probe the interfacial charge-transfer and separation behavior [40,41]. The Nyquist plot of **1**-SP under illumination displays a markedly smaller arc radius than that recorded in the dark, indicating a significant reduction in interfacial charge-transfer resistance upon light excitation [38,42,43]. These results corroborate the photocatalytic activity of complex **1**.

### 2.5. Photocatalytic Degradation Performance and Mechanism of MB

The photocatalytic degradation of organic dyes by complex **1** was investigated. As shown in Figure S7, the degradation rate of MB reached 83.8% within 180 min, whereas that of rhodamine B (RhB) and methyl orange (MO) remained significantly lower. Consequently, MB was selected as the model pollutant for subsequent photocatalytic studies.

**Figure 5:**
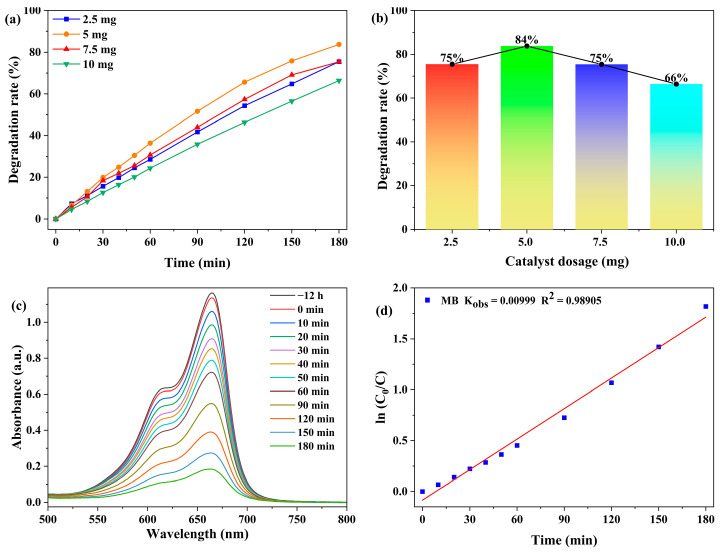
(**a**) Degradation rate of MB as a function of time under different catalyst dosages (reaction conditions: 20 ppm MB, 50 mL solution). (**b**) Degradation rates versus catalyst dosage after 180 min. (**c**) Absorption spectra of MB. (**d**) Pseudo-first-order kinetic fitting for the photocatalytic degradation of MB by **1**.

The effect of photocatalyst dosage (2.5, 5.0, 7.5, and 10.0 mg) on the degradation of MB (20 ppm, 50 mL) was examined. As shown in Figure 5 and Figure S8, when the dosage increased from 2.5 mg to 5.0 mg, the degradation rate of MB increased from 75.5% to 83.8%. This can be attributed to the fact that more catalyst dosage has more surface active sites [44]. However, the degradation rate decreased to 66.4%, when the dosage was further increased to 10.0 mg. This decline is attributed to the increased turbidity generated by the excess catalyst, which leads to significant light scattering and hinders the effective photon transport in the reaction system, thereby reducing the photocatalytic efficiency [44,45,46,47]. Accordingly, 5.0 mg was selected as the optimal catalyst dosage in the subsequent experiments.

The photocatalytic degradation of MB was modeled using the Langmuir-Hinshelwood kinetics. The kinetic equation is expressed as ln(C_0_/C) = kt, where C_0_ denotes the MB concentration at the initial stage of the photocatalytic reaction (t = 0), and C represents the MB concentration at reaction time t [21].

As shown in Figure 5d, in 180 min, the degradation of MB with an initial concentration of 20 ppm using 5.0 mg of catalyst exhibited kinetics that well followed a pseudo-first-order model with a calculated rate constant of 9.99 × 10^−3^ min^−1^.

**Figure 6:**
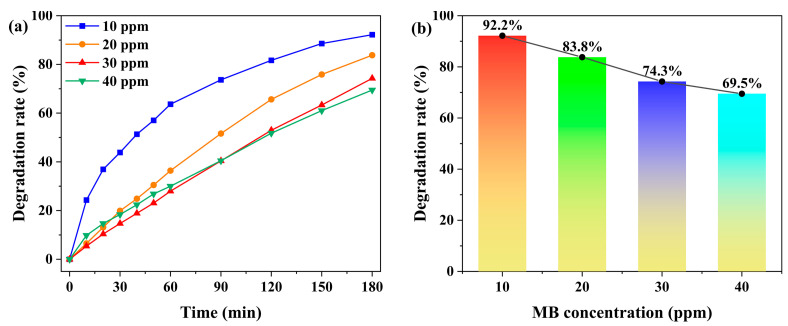
(**a**) Time-dependent degradation rates of MB under different initial concentrations (10 ppm, 20 ppm, 30 ppm, and 40 ppm), with the catalyst dosage fixed at 5.0 mg. (**b**) Degradation rates of MB under different initial concentrations after 180 min.

With a catalyst loading of 5.0 mg, the effect of initial MB concentration (10, 20, 30, and 40 ppm) on the degradation efficiency was investigated. As shown in Figure 6 and Figure S9, the degradation rate decreased from 92.2% to 69.5% as the MB concentration increased, demonstrating distinct concentration-dependent behavior. This trend is attributed to the competitive occupation of catalytic active sites by abundant MB molecules at higher concentrations, coupled with reduced solution transmittance that attenuates photon flux, collectively leading to diminished photocatalytic efficiency [48]. Considering both experimental observability and degradation performance, all subsequent photocatalytic experiments were conducted at an MB concentration of 20 ppm.

**Figure 7:**
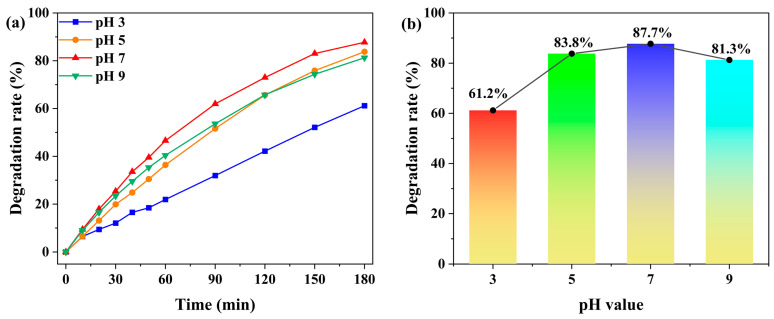
(**a**) Time-dependent degradation rates of MB under different solution pH values (pH 3, 5, 7, and 9), with the catalyst dosage of 5.0 mg and the initial MB concentration of 20 ppm. (**b**) Degradation rates of MB under different solution pH values (pH 3, 5, 7, and 9) after 180 min.

The effect of solution pH on the photocatalytic degradation of MB by complex **1** was systematically investigated (Figure 7 and Figure S10). Under constant conditions of 5.0 mg catalyst and 20 ppm MB (50 mL), the degradation rate increased from 61.2% to 87.7% as the pH was raised from 3 to 7. In acidic environments, the catalyst surface became protonated, generating strong electrostatic repulsion against cationic MB molecules and resulting in the lowest degradation efficiency at pH 3 [46,49]. As the pH increases, the extent of protonation decreases, leading to enhanced electrostatic attraction and improved MB adsorption. The efficiency reaches its maximum at neutral pH 7 [46,49]. However, when the pH was further increased to 9, the degradation rate decreased to 81.3%, primarily due to the excessive adsorption of dye molecules that occupied the active sites and consequently inhibited the catalytic reaction [46]. Therefore, pH 7 was determined to be the optimal value for this photocatalytic system. Meanwhile, we compared the prepared photocatalyst with the previously reported photocatalysts and found that it exhibits good photocatalytic degradation performance. For instance, the CPs prepared by Lin Du et al., Ali Ahmad et al., and Lei Li et al. achieved MB photocatalytic degradation rates of 59.92%, 86.6%, and 96.8% (the highest efficiency was attributed to the addition of H_2_O_2_), respectively [50,51,52].

**Figure 8:**
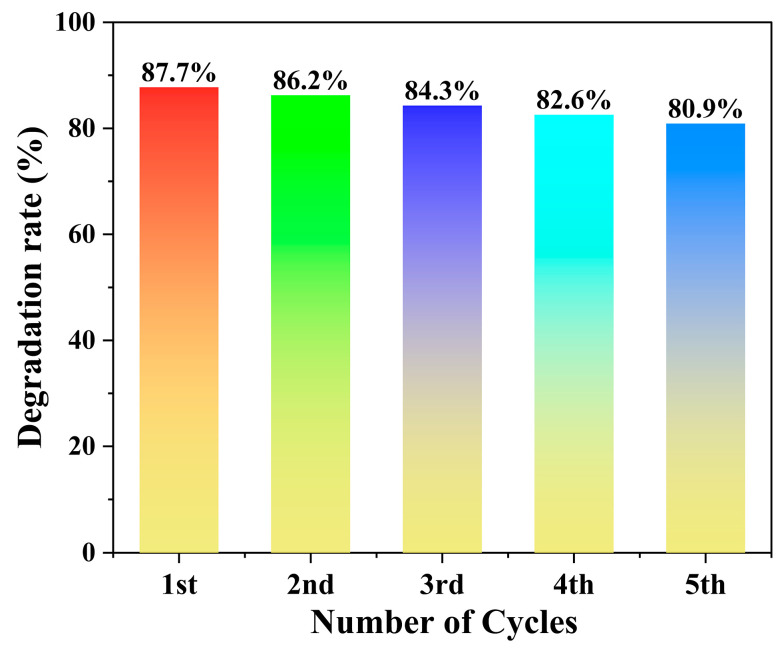
Cyclic stability of the photocatalyst.

As shown in Figure 8, the cyclic stability tests revealed no significant decline in catalytic performance over five consecutive cycles, indicating the excellent stability of the catalyst.

**Figure 9:**
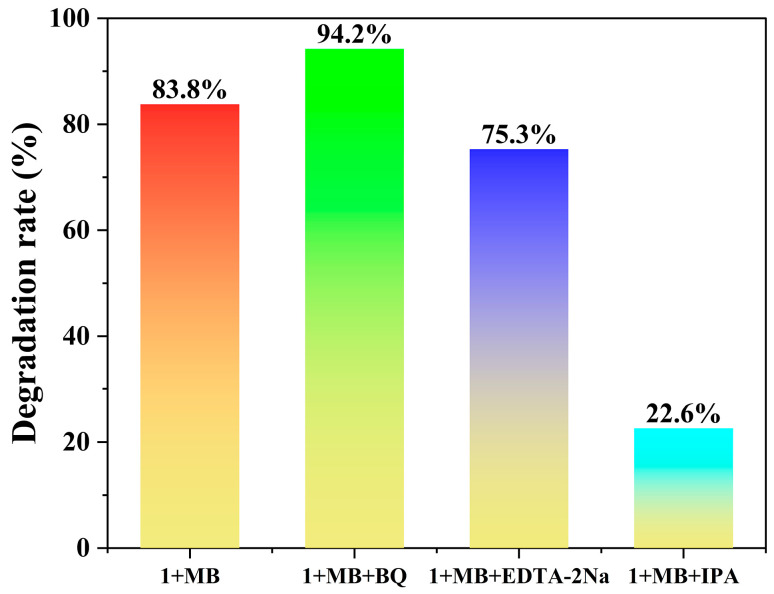
Photocatalytic degradation of MB solutions (20 ppm, 50 mL) with and without scavengers (using 5.0 mg of **1**).

To identify the crucial active species in the process of photocatalytic degradation, benzoquinone (BQ), disodium ethylenediaminetetraacetate (EDTA-2Na), and isopropanol (IPA) were employed as scavengers for ·O_2_^−^, h^+^, and ·OH, respectively [21,53]. As shown in Figure 9, the introduction of EDTA-2Na and IPA significantly suppressed the degradation efficiency, reducing it to 75.3% and 22.6%, respectively. The result indicated that both h^+^ and ·OH participate in the reaction, with ·OH being the dominant active species. In contrast, the degradation efficiency increased to 94.2% with the introduction of BQ. This enhancement is due to the dual function of BQ in scavenging ·O_2_^−^ while simultaneously suppressing e^−^/h^+^ recombination [54].

**Figure 10:**
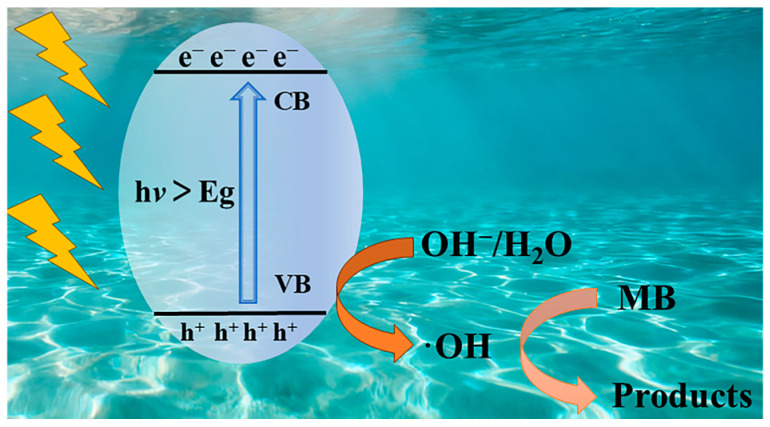
The schematic mechanism diagram of **1** photocatalytic degradation of MB.

Complex **1** exhibits p-type semiconductor characteristics with h^+^ as the majority carriers [35]. The probably degradation mechanism is described in Figure 10. Under Xe lamp irradiation, the photocatalyst undergoes photoexcitation to generate electron-hole pairs (e^−^/h^+^), where e- are excited from the valence band (VB) to the conduction band (CB), then h^+^ preferentially reacts with H_2_O or OH^−^ to produce ·OH [55]. This pathway proves more efficient than direct oxidation of pollutants, thereby significantly enhancing the photocatalytic performance of the system.

### 2.6. Electrochemical Properties

#### Cyclic Voltammetric Behaviors

**Figure 11:**
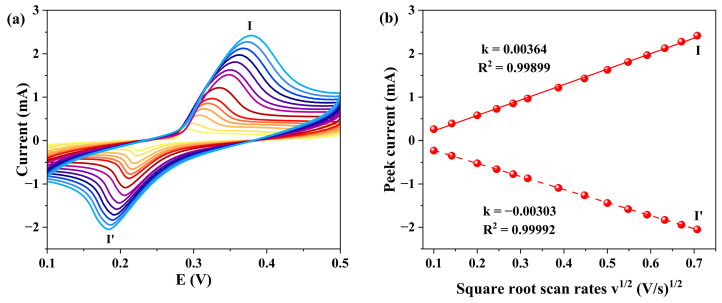
(**a**) CV curves of **1**-GCE (scan rates from inner to outer: 0.01 0.02, 0.04, 0.06, 0.08, 0.1, 0.15, 0.25, 0.3, 0.35, 0.4, 0.45, and 0.5 V s^−1^). (**b**) Relationship between the peak currents and scan rates.

The electrochemical performance of **1**-GCE was studied by cyclic voltammetry (CV) in 1 M KOH aqueous solution at different scan rates. As shown in Figure 11, a pair of quasi-reversible redox peaks (I-I′) in the potential range of 0.1–0.5 V. The half-wave potential E_1/2_ was 0.261 V at a scan rate of 0.02 V s^−1^ (E_1/2_ = (E_pa_ + E_pc_)/2, where E_pa_ and E_pc_ represent the anodic and cathodic peak potentials, respectively). The redox peaks (I-I′) can be attributed to the redox behavior of the metal center. The anodic process (peak I) corresponds to the oxidation reaction: Ni^2+^ → Ni^3+^ + e^−^, while the cathodic process (peak I′) corresponds to the reduction reaction: Ni^3+^ + e^−^ → Ni^2+^ [19,56,57]. As the scan rates increased from 0.01 V s^−1^ to 0.5 V s^−1^, the anodic peak potential shifted positively, while the cathodic peak potential shifted negatively. The peak currents showed a linear relationship with the square root of the scan rate (v^1/2^), suggesting a diffusion-controlled electrode process [58].

### 2.7. Electrochemical Detection of NOR

#### 2.7.1. The Amperometric Detection of NOR

**Figure 12:**
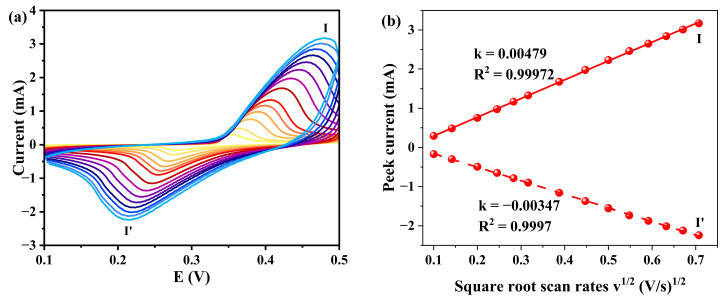
(**a**) CVs of **1**-GCE in a 0.5 M KOH containing 33 μM NOR with different scan rates (scan rates from inner to outer: 0.01 0.02, 0.04, 0.06, 0.08, 0.1, 0.15, 0.2, 0.25, 0.3, 0.35, 0.4, 0.45, and 0.5 V s^−1^). (**b**) Relationship between the peak currents and scan rates.

The electrochemical behavior of the **1**-GCE toward 33 μM norfloxacin (NOR) was investigated in a KOH aqueous solution. As shown in Figure 12, a pair of redox peaks was observed at scan rates ranging from 0.01 to 0.5 V s^−1^, indicating a quasi-reversible electrochemical reaction of NOR on the electrode surface [59]. The E_1/2_ was approximately 0.319 V at a scan rate of 0.02 V s^−1^. Similar to the case in blank KOH solution, the electrode reaction process of **1**-GCE is also diffusion-controlled in KOH solution containing 33 μM NOR [60].

As seen in Figure S11, as the concentration of NOR increased, the anodic peak current gradually increased while the cathodic peak current decreased correspondingly, indicating that **1**-GCE exhibits electrocatalytic oxidation activity toward NOR [19].

#### 2.7.2. The DPV Detection of NOR

Accordingly, differential pulse voltammetry (DPV) was employed to evaluate the sensing performance of **1**-GCE for NOR detection. As shown in Figure S12, no DPV response for NOR was observed at the bare electrode, indicating that the electrode itself does not cause interference.

**Figure 13:**
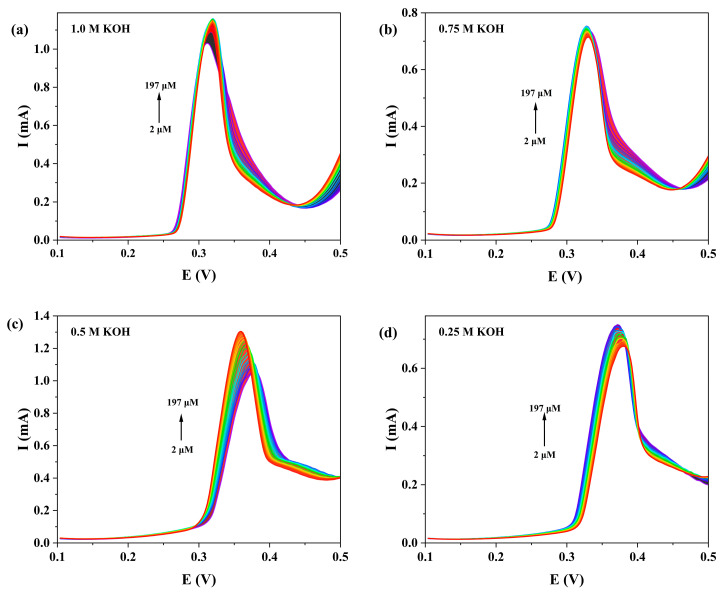
DPV responses of 1-GCE to NOR in the concentration range of 2~197 μM under different concentrations of KOH aqueous solution (1 M (**a**), 0.75 M (**b**), 0.5 M (**c**), and 0.25 M (**d**)).

**Figure 14:**
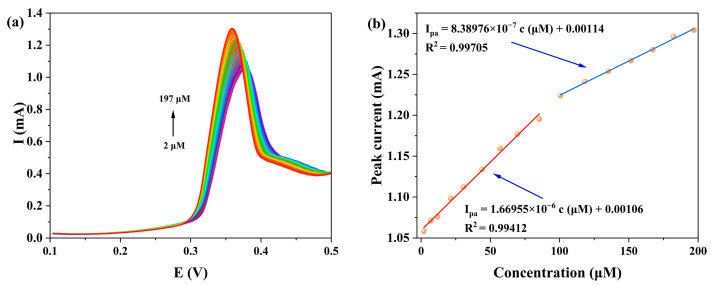
(**a**) The DPV responses of **1**-GCE to different concentrations of NOR in a 0.5 M KOH aqueous solution. (**b**) The corresponding linear plot between NOR concentrations and anodic peak currents (Red line: 2–85 μM (Ipa = 1.72187c + 9.87987, R2 = 0.99006); Blue line: 100–197 μM (Ipa = 0.83898c + 94.16017, R2 = 0.99705)).

We systematically investigated the influence of KOH concentration (0.25, 0.5, 0.75 and 1.0 M) on the DPV detection of NOR at the **1**-GCE (Figure 13). The results showed that **1**-GCE exhibited the most sensitive DPV response toward NOR in 0.5 M KOH. Under this optimal condition, as shown in Figure 14, the anodic peak current increased gradually with increasing NOR concentration, showing good linear relationships in two concentration ranges: 2–85 μM (I_pa_ = 1.72187c + 9.87987, *R*^2^ = 0.99006) and 100–197 μM (I_pa_ = 0.83898c + 94.16017, *R*^2^ = 0.99705) [61,62,63]. The limit of detection (LOD) for NOR at the **1**-GCE was calculated to be 1.74 μM based on the 3σ criterion (where σ is the standard deviation of the current response of the electrode in blank KOH solution, *n* = 15, S/N = 3) [62]. The above results demonstrate that the constructed **1**-GCE sensor exhibits good sensitivity toward NOR.

**Figure 15:**
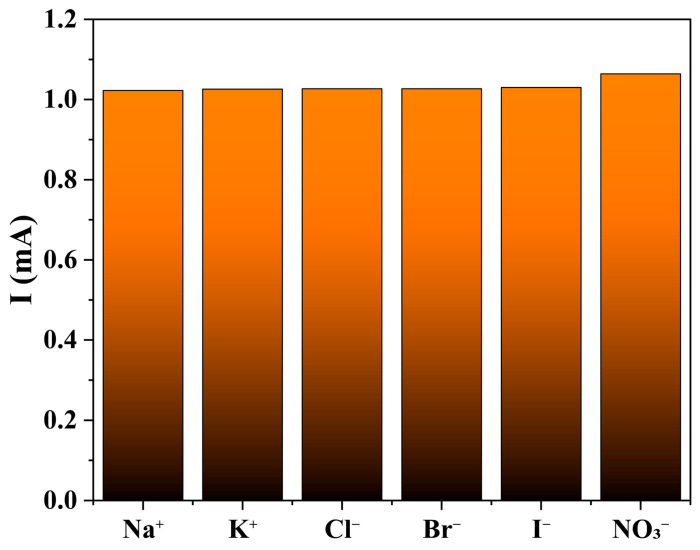
The influence for NOR (33 μM) from following interfering ions (99 mM): Na^+^, K^+^, Cl^−^, Br^−^, I^−^ and NO_3_^−^.

The selectivity of the **1**-GCE sensor was evaluated by examining the effect of seven common interfering ions (Na^+^, K^+^, Cl^−^, Br^−^, I^−^, and NO_3_^−^, each at 99 mM) in a 0.5 M KOH solution containing 33 μM NOR [62,63]. The results indicated that none of these ions caused significant interference in the detection of NOR (Figure 15).

The performance of the prepared **1**-GCE sensor was compared with that of other reported electrochemical sensors. As shown in Table 1, the **1**-GCE sensor possesses a wider linear range for the detection of NOR, although the LOD of **1**-GCE is not low.

## 3. Experimental Section

### 3.1. Materials and Methods

All starting materials were commercially available, used directly without further purification. Among them, H_2_L (H_2_L = 4,4′-(1H,1′H-[2,2′-biimidazole]-1,1′-diyl)dibenzoic acid) was purchased from Jilin Chinese Academy of Sciences—Yanshen Technology Co., Ltd. (Changchun, China), with a purity of 97%. Infrared (IR) spectra (KBr pellets) were recorded on a NICOLET6700 FT-IR spectrometer (Waltham, MA, USA) at room temperature in the range of 400–4000 cm^−1^. Thermogravimetric analysis (TGA) was performed on a NETZSCH (Selb, Germany) STA 449F3 instrument, heating from room temperature to 900 °C in air at a heating rate of 10 °C·min^−1^. Powder X-ray diffraction (PXRD) pattern was collected on a Bruker (Billerica, MA, USA) D8 Advance X-ray diffractometer (graphite-monochromated Cu-*Kα*1, *λ* = 1.5406 Å) with a 2θ range of 5–50° and a step size of 0.02°. Electrochemical behaviors were tested on a CHI760E electrochemical workstation (Shanghai, China). Photocatalytic performance was conducted using a CEL-HXF300A Xenon lamp light source (Beijing, China). UV-Vis spectra were recorded on a TU-1950 spectrophotometer. The UV-Vis diffuse-reflectance spectroscopy (UV-Vis DRS) was characterized using a SHIMADZU (Tokyo, Japan) UV-2600i UV-Vis-NIR spectrophotometer with barium sulfate (BaSO_4_) as the reference substance. Elemental analyses for C, H, and N were determined on a Perkin-Elmer (Waltham, MA, USA) 2400 Elemental Analyzer.

### 3.2. Syntheses of [Ni(L)(H_2_O)_3_]_n_·nH_2_O *(**1**)*

NiCl_2_·6H_2_O (0.0240 g, 0.10 mmol), H_2_L (0.0370 g, 0.15 mmol), and 6 mL H_2_O were mixed and stirred for 30 min. The pH value of the mixture was adjusted to 6.27 (±0.5) with 1.0 mol·L^−1^ HCl and NaOH, and the mixture was further stirred for another 30 min. Subsequently, the mixture was transferred into a 12 mL Teflon-lined autoclave, sealed in an oven, and heated at a constant temperature of 170 °C for 96 h. The mixture was then cooled down naturally to room temperature, and purple-gray square block crystals of complex **1** were obtained. Yield 55% (based on Ni). Anal. Calc. for C_20_H_20_N_4_NiO_8_, C: 47.75; H: 4.01; N: 11.14 (%). Found, C: 47.20; H: 4.28; N: 11.08 (%). IR (KBr, cm^−1^): 3230 m, 1606 s, 1563 s, 1508 m, 1394 s, 1332 m, 1140 m, 509 w, 418 w.

### 3.3. X-Ray Crystallography

The single-crystal X-ray diffraction data for complex **1** were collected on a Bruker APEX CCD diffractometer with Mo *Kα* radiation (*λ* = 0.71073 Å, graphite monochromator) at 298 K. The crystal structure was solved by direct methods and refined with the Olex2 program package using full-matrix least-squares minimization based on *F*^2^. All non-hydrogen atoms were refined with anisotropic displacement parameters. Hydrogen atoms attached to ligands were placed theoretically. The detailed crystallographic parameters are summarized in Table S1. Selected bond lengths and angles are provided in Table S2. The CCDC deposition number for the crystal is 2,494,941.

### 3.4. Photocatalytic Experiments

The photocatalytic activity of complex **1** for degrading organic dyes was investigated. The photocatalyst was dispersed in the organic dye solution (50.0 mL) and kept in the dark for 12 h to reach adsorption–desorption equilibrium. Subsequently, 1.5 mL of the suspension was withdrawn, filtered to remove the solid catalyst, and subjected to UV-vis measurement to obtain the initial absorbance. The remaining mixture was then irradiated under continuous stirring with full-spectrum light from a 300 W Xe lamp (Beijing, China). At selected time intervals, 1.5 mL aliquots were collected, filtered, and analyzed by UV-vis spectroscopy to monitor the degradation progress.

Furthermore, the degradation kinetics were studied using a pseudo-first-order kinetic model (expressed as $ln(\frac{C_{0}}{C})=k_{obs}\times t$, where *C*_0_ is the initial concentration, *C* is the concentration at time *t*, and *k_obs_* is the apparent rate constant).

### 3.5. Preparation of the Working Electrode

#### 3.5.1. Preparation of Glassy Carbon Electrode

The glassy carbon electrode modified with **1** (**1**-GCE) was prepared as follows. A glassy carbon electrode (GCE, 3 mm in diameter) was polished on a polishing pad with 0.3 and 0.05 μm alumina powders and then air-dried. Complex **1** and acetylene black were mixed in a 1:1 mass ratio by grinding. The resulting mixture was dispersed in 330 μL deionized water, 165 μL isopropyl alcohol, and 10 μL 5% Nafion solution, and sonicated for 120 min to obtain a uniform dispersion. Finally, 5 μL of the dispersion was dropped onto the surface of the GCE and allowed to dry in air before testing.

A conventional three-electrode system was employed, with the working electrode (**1**-GCE), the auxiliary electrode (platinum sheet), and the reference electrode (Hg/Hg_2_Cl_2_).

The DPV measurements were performed in a potential of 0.1 to 0.5 V at a scan rate of 0.02 V s^−1^, with pulse parameters of 0.05 V amplitude, 0.05 s width, and 0.2 s interval.

#### 3.5.2. Preparation of Semiconductor Photoelectrode.

For the photoelectrochemical tests, the semiconductor photoelectrode (**1**-SP) was fabricated by drop-casting 30 μL of the pre-prepared dispersion onto a 1 × 1 cm^2^ ITO glass, which had been ultrasonically cleaned in absolute ethanol for 30 min, rinsed with deionized water, and dried. The resulting **1**-SP was then dried at 60 °C for 1 h.

## 4. Conclusions

In this study, a new nickel-based coordination polymer, [Ni(L)(H_2_O)_3_]_n_·nH_2_O, has been hydrothermally assembled from H_2_L (H_2_L = 4,4′-(1H,1′H-[2,2′-biimidazole]-1,1′-diyl)dibenzoic acid). Single-crystal X-ray diffraction reveals that Ni^2+^ centers are connected by L^2−^ to generate 1D Z-shaped chains, which are further extended into a 3D supramolecular network through hydrogen-bonding interactions. Photocatalytic performance studies demonstrate that complex **1** exhibits photocatalytic activity in the degradation of MB. Under the conditions of 5 mg of catalyst, 20 ppm initial MB concentration, and pH 7, a degradation efficiency of 87.7% is achieved after 180 min. In addition, **1**-GCE displayed electrochemical sensing performance for the detection of NOR via DPV. Within the concentration range of 2–197 μM, the current response showed a good linear relationship with NOR concentration, with a detection limit as low as 1.74 μM (S/N = 3), indicating its promising potential for trace NOR detection. These results demonstrate that complex **1** concurrently functions as an efficient photocatalyst for organic-dye degradation and as a sensitive electrochemical sensor for trace norfloxacin NOR, substantiating its potential as a dual-purpose material for the treatment of complex pollutants.

## Figures and Tables

**Table 1:** Comparison of the performance of different modified electrodes for NOR detection.

Detection Techniques	Active Electrode Materials	Line Arrange (μM)	Detection Limit (μM)	References
DPV	CuO/MWCNTs	1~47.7	0.321	[64]
DPV	Pd^2+^@P-CDP/COFs	0.08~100	0.031	[65]
DPV	MWCNTs-TOCT	0.5~8	0.1	[66]
DPV	**1**-GCE	2~197	1.74	This work

## Data Availability

Data are contained within the article and Supplementary Materials.

## Supplementary Materials

The following supporting information can be downloaded at: https://www.mdpi.com/article/10.3390/molecules30224366/s1, Figure S1: The coordination modes of the organic ligand in this work (**a**) and (**b**), and in the reported reference literature (**c**). Figure S2: PXRD pattern of **1**. Figure S3: Infrared spectra of H_2_L ligand (**a**) and **1** (**b**). Figure S4: TG and DSC curves of **1**. Figure S5: (**a**) The band gap energy of **1**, calculated using the Kubelka-Munk formula, is 3.3362 eV, with the corresponding solid-state UV-visible absorption spectrum shown in the inset. (**b**) The Mott-Schottky plot of the **1**-GCE electrode was measured at a frequency of 1000 Hz. The flat-band potential is determined from the intersection of the extrapolated dashed lines. Figure S6: (**a**) Photocurrent response of **1**-SP. (**b**) EIS Nyquist plots of the **1**-SP in 1 mol/L KOH solution under visible light irradiation. Figure S7: Photocatalytic degradation of MB, MO, and RhB (20 ppm each) using **1** as catalyst. Reaction conditions: catalyst dosage 5 mg, solution volume 50 mL, 300 W Xe lamp, illumination time 180 min. Figure S8: Absorption spectra of **1** (2.5 mg (**a**), 5.0 mg (**b**), 7.5 mg (**c**), and 10.0 mg (**d**)) for the MB solutions (20 ppm and 50 mL) under 300 W Xe light with full spectrum. Figure S9: Photocatalytic degradation profiles of **1** for MB solutions (initial concentration: 10 ppm (**a**), 20 ppm (**b**), 30 ppm (**c**), and 40 ppm (**d**)) (The dosage of **1** was 5.0 mg). Figure S10: Photocatalytic degradation profiles of **1** for MB solutions (pH 3 (**a**), pH 5 (**b**), pH 7 (**c**), and pH 9 (**d**)) (The dosage of **1** was 5.0 mg, and the initial concentration of MB was 20 ppm). Figure S11: The CV cures of **1**-GCE in 0.5 M KOH aqueous solution containing 0, 2, 4, 6, 8 and 10 mM NOR (0.2 V s^−1^). Figure S12: The DPV response of 33 μM NOR in a 0.5 M KOH solution using a bare- and **1**-GCEs at a scan rate of 0.02 V s^−1^. Table S1: Crystal data and structure refinements for complex **1**. Table S2: Selected bond lengths (Å) and bond angles (°) for complex **1**.
