# Supplementary material for: A 1D [Ni(L)(H2O)3]n·nH2O Coordination Polymer as a Dual Function Material for Antibiotic Detection and Dye Photo-Degradation
Source: Molecules. 2025 Nov 12;30(22):4366. doi: 10.3390/molecules30224366 (PMC12655596; doi:10.3390/molecules30224366)
Supplement: Supplementary file 1 [file molecules-30-04366-s001.zip › molecules-3959671-supplementary.pdf]

Supplement files

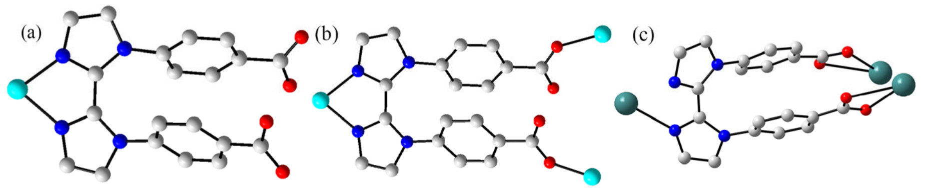

Figure S1. The coordination modes of the organic ligand in this work (a) and (b), and in the reported reference literature (c) [1].

[1] Liu, X.; Cai, H.; Zhou, R.; Li, Y. Construction of a PbII Coordination Polymer From a Semi-rigid Ditopic 2,2-biimidazole Derivative: Synthesis, Crystal Structure and Characterization. Acta Crystallogr C Structural Chemistry 2023, 79, 263-268, doi:10.1107/S2053229623004783.

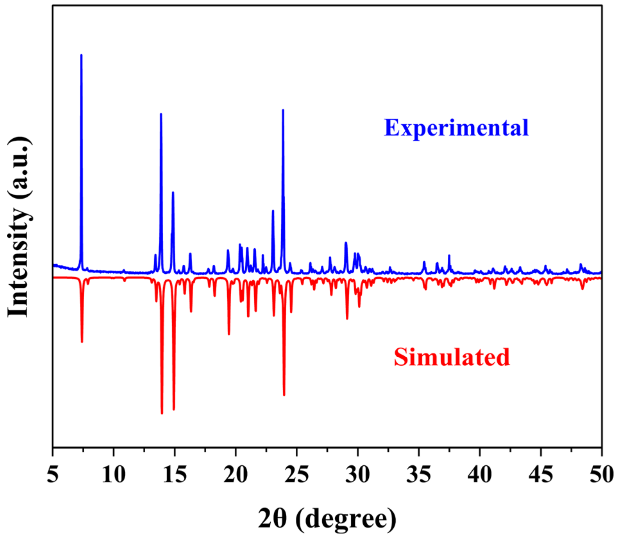

Figure S2. PXRD pattern of **1**.

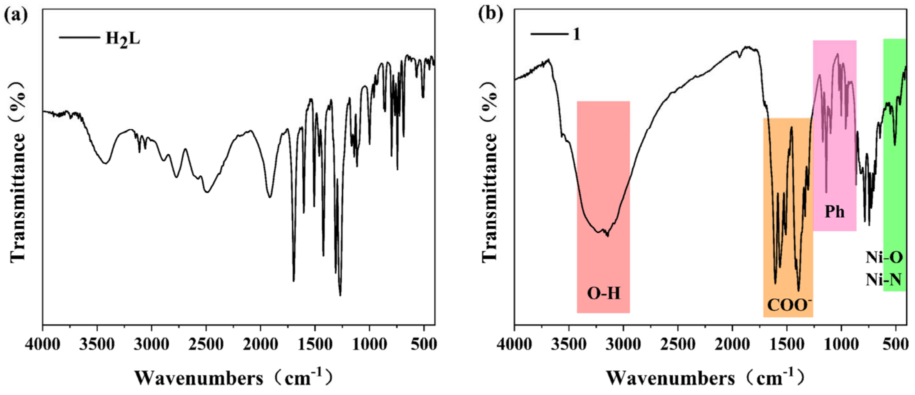

Figure S3. Infrared spectra of H_2L ligand (a) and **1** (b).

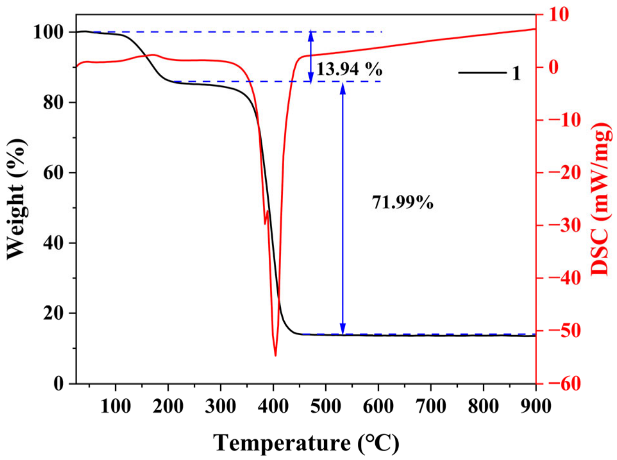

Figure S4. TG and DSC curves of **1**.

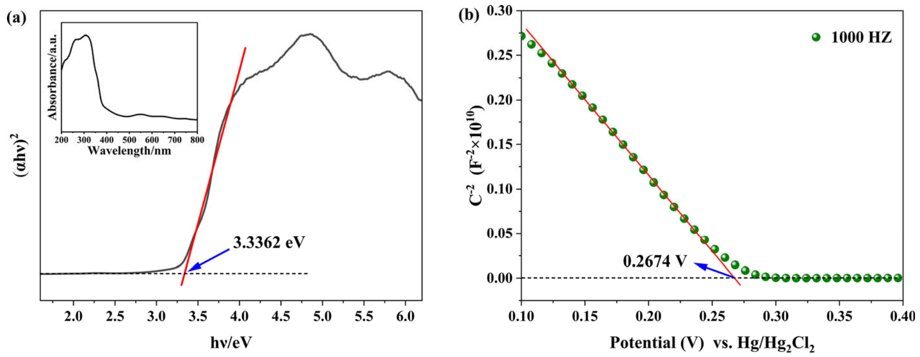

Figure S5. (a) The band gap energy of **1**, calculated using the Kubelka-Munk formula, is 3.3362 eV, with the corresponding solid-state UV-visible absorption spectrum shown in the inset. (b) The Mott-Schottky plot of the **1**-GCE electrode was measured at a frequency of 1000 Hz. The flat-band potential is determined from the intersection of the extrapolated dashed lines.

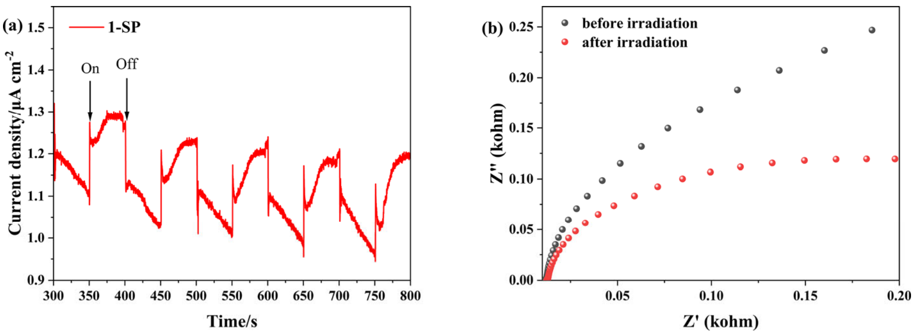

Figure S6. (a) Photocurrent response of **1**-SP. (b) EIS Nyquist plots of the **1**-SP in 1 mol/L KOH solution under visible light irradiation.

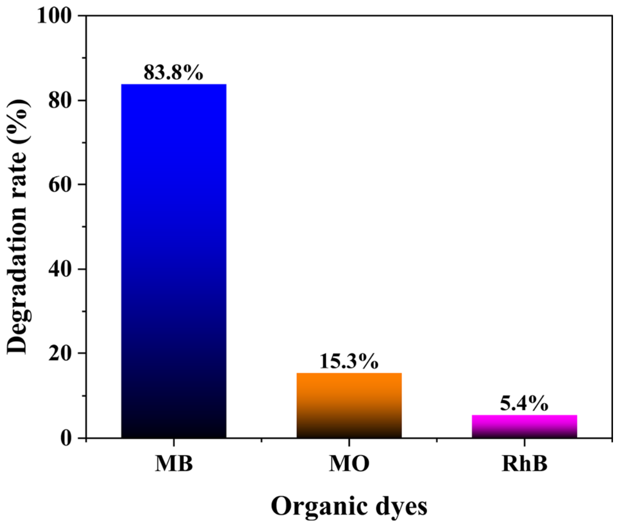

Figure S7. Photocatalytic degradation of MB, MO, and RhB (20 ppm each) using **1** as catalyst. Reaction conditions: catalyst dosage 5 mg, solution volume 50 mL, 300 W Xe lamp, illumination time 180 min.

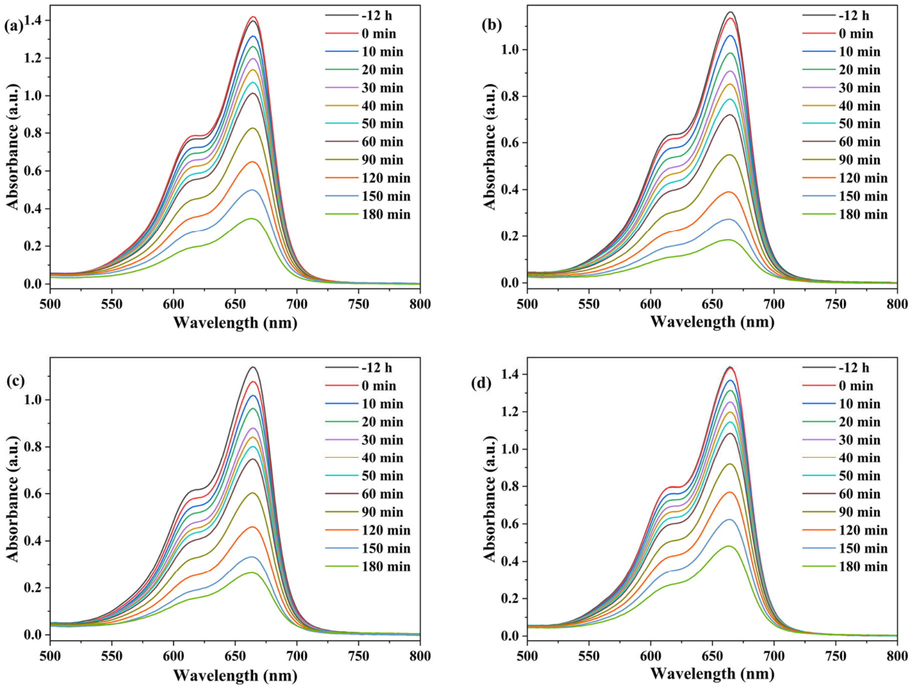

Figure S8. Absorption spectra of **1** (2.5 mg (a), 5.0 mg (b), 7.5 mg (c), and 10.0 mg (d)) for the MB solutions (20 ppm and 50 mL) under 300 W Xe light with full spectrum.

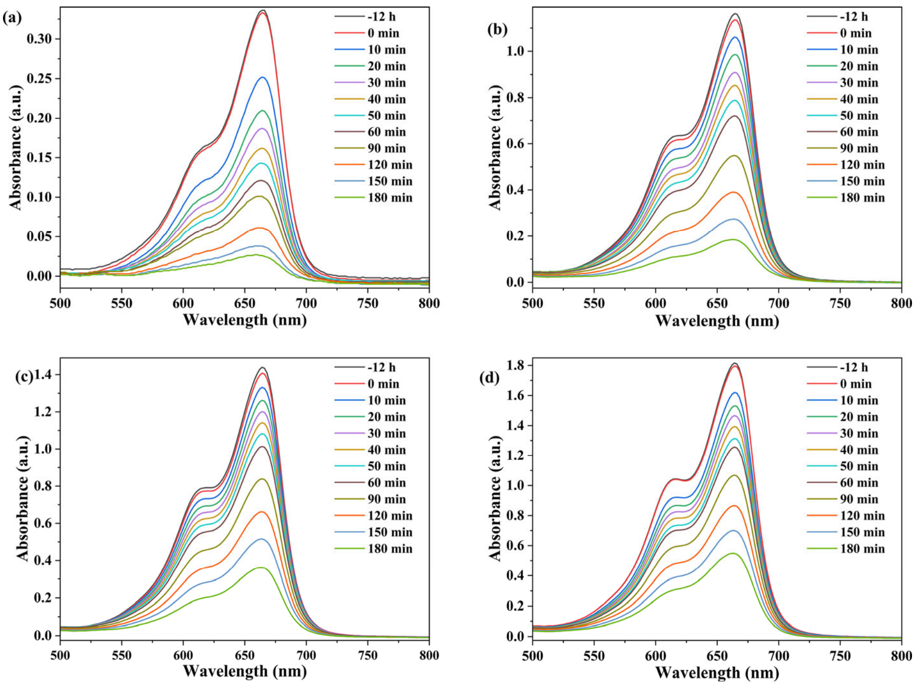

Figure S9. Photocatalytic degradation profiles of **1** for MB solutions (initial concentration: 10 ppm (a), 20 ppm (b), 30 ppm (c), and 40 ppm (d)) (The dosage of **1** was 5.0 mg).

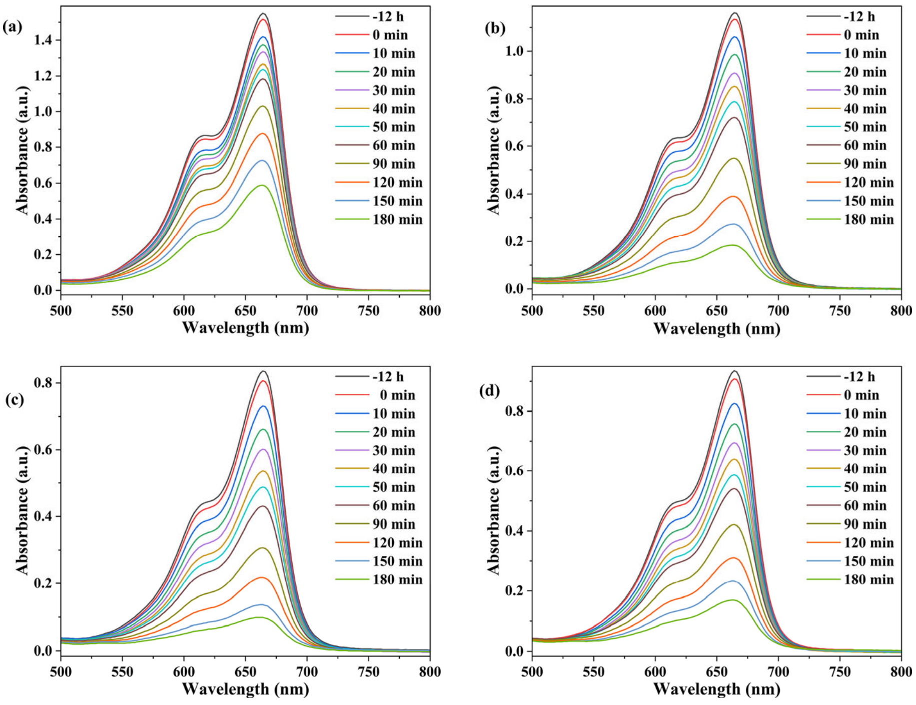

Figure S10. Photocatalytic degradation profiles of **1** for MB solutions (pH 3 (a), pH 5 (b), pH 7 (c), and pH 9 (d)) (The dosage of **1** was 5.0 mg, and the initial concentration of MB was 20 ppm).

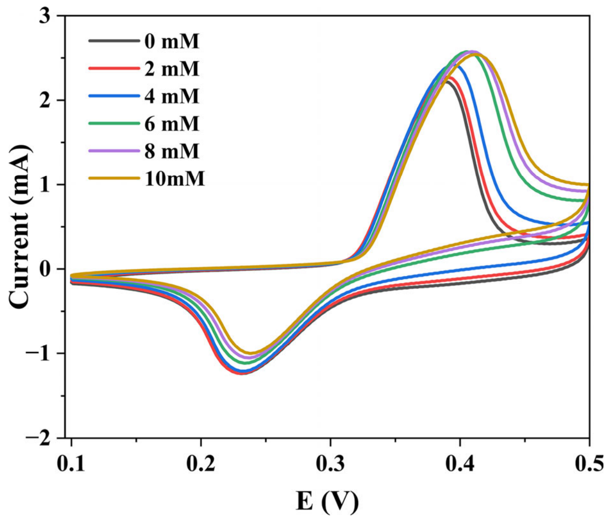

Figure S11. The CV cures of **1**-GCE in 0.5 M KOH aqueous solution containing 0, 2, 4, 6, 8 and 10 mM NOR (0.2 V s^{-1}).

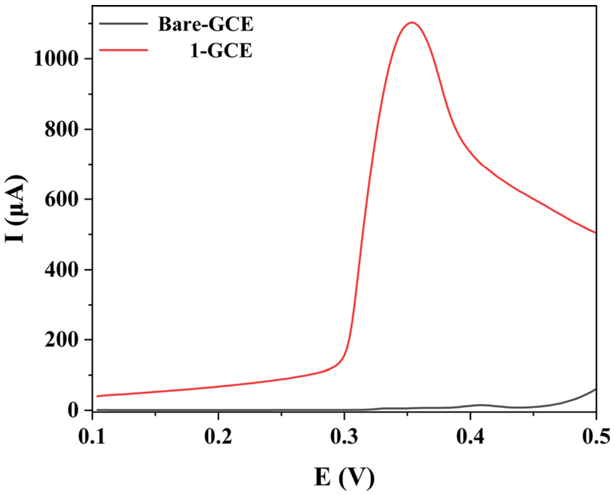

Figure S12. The DPV response of 33 μM NOR in a 0.5 M KOH solution using a bare- and **1**-GCEs at a scan rate of 0.02 V s^{-1}.

Table S1. Crystal data and structure refinements for complex **1**.

	**1**
Formula	$C_{20}H_{20}N_4NiO_8$
Mol. wt.	503.11
Crystal system	Monoclinic
Space group	*C2/c*
a (Å)	12.7695(3)
b (Å)	23.8729(6)
c (Å)	13.2016(3)
α (°)	90
β (°)	96.6630(10)
γ (°)	90
V (Å^3)	3997.26(17)
Z	8
Dc (g cm^{-3})	1.031
μ (mm^{-1})	1.672
F (000)	2080
Reflection collected	44947
R_{int}	0.683
GOF	0.999
Final R^{ab} indices [I>2σ(I)]	R_1 = 0.627; wR_2 = 0.683
R indices (all data)	R_1 = 0.637; wR_2 = 0.746

[a] $R_1 = \Sigma||Fo| - |Fc||/\Sigma|F|$; [b] $wR_2 = |\Sigma w (|Fo|^2 - |Fc|^2)^2/\Sigma|w(F)^2|^{1/2}$.

Table S2. Selected bond lengths (Å) and bond angles (°) for complex **1**.

		**1**	
Ni1-O5	2.097(2)	Ni1-N1	2.100(2)
Ni1-N3	2.071(2)	Ni2-O3	2.041(2)
Ni2-O6	2.074(3)	Ni2-O7	2.061(2)
O5-Ni1-O5	179.02(11)	O5-Ni1-N1	91.69(9)
N3-Ni1-O5	90.84(8)	N1-Ni1-N1	78.94(12)
N3-Ni1-N3	78.18(12)	N3-Ni1-N1	179.61(9)
O3-Ni2-O6	91.54(13)	O3-Ni2-O3	180
O6-Ni2-O6	180	O3-Ni2-O7	94.00(9)
O7-Ni2-O6	91.63(11)	O7-Ni2-O7	180
